# Theorie und Phänomenologie des Abjekts

**DOI:** 10.1007/s43638-021-00026-8

**Published:** 2021-12-10

**Authors:** Larissa Pfaller, Michael Schetsche

**Affiliations:** 1 Institut für Soziologie, Friedrich-Alexander-Universität Erlangen-Nürnberg, Kochstr. 4, 91054 Erlangen, Deutschland; 2 Institut für Grenzgebiete der Psychologie und Psychohygiene e.V., Wilhelmstraße 3a, 79098 Freiburg i. Br., Deutschland

‚Abjekt‘ ist das erste Lemma im *Lexikon der Postmoderne* (Wolf 2010). Damit hat es, obschon die Reihung freilich der alphabetischen Ordnung geschuldet ist, doch auch einen seiner theoretisch-konzeptionellen Bedeutung entsprechenden Platz gefunden. Abjektion, das ist nicht nur der erste Schritt zur Subjektwerdung, sondern auch der erste Schritt zur Konstitution einer kulturellen Ordnung und gleichzeitig der grundlegende Mechanismus zu deren Aufrechterhaltung. So jedenfalls beschreibt es die bulgarisch-französische Philosophin, Psychoanalytikerin und Literaturtheoretikerin Julia Kristeva. Mit *Powers of Horror. An Essay on Abjection* (1982) hat sie den Konzepten *abject* und *abjection* nicht nur zu einer breiteren wissenschaftlichen Rezeption verholfen, sondern auch ein bedeutendes theoretisches Instrumentarium zur Analyse der psychosozialen Verfasstheit der heutigen Kultur geschaffen. Denn die psychische Konstitution des Subjekts und die Institution der kulturellen Ordnung sind untrennbar verwoben, ja mehr noch: sie sind zwei Seiten ein- und desselben Mechanismus, zwei Seiten einer Medaille. Ist der Status des Subjekts gefährdet, ist damit gleichzeitig das Fundament der modernen Gesellschaft infrage gestellt. Werden kulturell etablierte Grenzen überschritten, wird auch die psychische Integrität des Subjektes bedroht.

Kristeva leitet diesen Zusammenhang aus der Anlage der frühkindlichen Entwicklung und den grundlegenden Mechanismen der Konstitution von Kultur, der gesellschaftlichen Logik elementarer Unterscheidungen, ab: In der frühesten Entwicklungsphase existiert für die psychische Monade kein Unterschied zwischen dem eigenen Selbst und der Welt. Vielmehr herrscht eine unspezifische und undifferenzierte Vielheit an Bedürfnissen, Wahrnehmungen und Gefühlen. Damit so etwas wie eine Selbstwerdung überhaupt erst beginnen und eine initiale Grenze zwischen Selbst und Umwelt gezogen werden kann, muss etwas aus der *chora*, dem undifferenzierten Urgrund, verworfen werden und dadurch zum Nicht-Selbst werden. Damit wird ein aversives Verhältnis zwischen dem Selbst und dem Verworfenen etabliert. In der Folge dieser initialen Selbstkonstitution müssen die Grenzen des Körpers und des Subjektes beständig aufrechterhalten und die Abjektion, die radikale Verwerfung von nun nicht (mehr) tolerierbaren Teilen des Selbst, beständig vollzogen werden. So ist schließlich der Umgang mit Schmutz oder Körperausscheidungen als elementare Kulturleistung zu verstehen, die beständig vollzogen werden muss, um die Ordnung des Alltagslebens aufrechterhalten zu können. Abjekt ist nicht nur der verworfene und objektivierte Selbstanteil des Subjektes, sondern alles, was die existenziellen Grenzen zwischen Subjekt und Objekt, Kultur und Natur bedroht. In der Abjektion ist die Konstitution des Subjekts elementar an die kulturelle Ordnung gebunden. Kristevas Konzept erlaubt es damit, nicht nur die subjektive Erfahrungsebene, sondern auch die normative Dimension der Abjektion in den Blick zu nehmen (vgl. Pfaller 2021).

Die Kulturwissenschaften und die Kunsttheorie haben das analytische Potenzial des Abjekts bereits seit längerem für sich entdeckt (Schmitz 2000, S. 37; Menninghaus 2003, S. 365) und mit der *Abject Art* nutzt eine ganze Kunstrichtung die vom Abjekten ausgehende Faszination (vgl. Zimmermann in dieser Ausgabe). Im Gegensatz dazu hat – mit wenigen Ausnahmen – gerade die deutschsprachige Soziologie bisher weder das theoretische Potenzial des Abjektbegriffs noch die empirischen Möglichkeiten einer Abjektanalyse für sich nutzbar gemacht. So wurde *abject* und *abjection* bei Übersetzungen ins Deutsche – wie beispielhaft die Schriften Judith Butlers (1993) zeigen – nicht etwa mit „Abjekt“ und „Abjektion“, sondern je nach Kontext mit „das Verworfene“, „Verworfenheit“, „Verwerflichkeit“ oder „Verwerflichmachung“ übersetzt, was erstens theoretische Anschlüsse an die Theorie der Abjektion schwer erkennbar macht und zweitens eine Etablierung der Begriffe im deutschsprachigen Raum erschwert hat (vgl. Pfaller 2021).

Das vorliegende *Special Issue* möchte daher das Potenzial von Abjekt und Abjektion im deutschsprachigen Raum ausloten. Hierzu versammelt es Forschungsperspektiven aus der Soziologie (Böhrer, Balke, Kotthaus) aber auch aus schon länger mit dem Abjekt vertrauten Disziplinen der Kunsttheorie (Zimmermann), Amerikanistik (Mueller, Berressem) und Psychologie (Straub) sowie der Philosophie (Schramm) und Palliativmedizin (Kurkowski und Heckel). Die Herausgeber*innen des vorliegenden *Special Issues* haben bereits erste Bemühungen für eine genuin soziologische Anwendung des Abjekts auch im deutschsprachigen Raum unternommen: Gemeinsam mit Martina Biebert übernimmt Michael Schetsche das Abjekt in das Begriffsinstrumentarium der Wissenssoziologie und bildet ein Konzept der „kulturellen Abjekte“. Neben orthodoxem und heterodoxem Wissen identifizieren die Autor*innen damit eine Form dauerhaft ausgeschlossenen Wissens, gleichsam „unsichtbare Wissensbestände“, die soziale Realität zwar prägen, auf die selbst aber nur über „Auslassungen“ und „Leerstellen“ im Diskurs geschlossen werden kann (Biebert und Schetsche 2016, S. 98). Larissa Pfaller verortet das Abjekt konzeptionell in der Theorie des sozialen Imaginären (Pfaller 2021) und macht einen ersten Vorschlag für eine Abjektanalyse, die nicht nur nach dem transgressiven Potenzial des Abjekts fragt (welche existenzielle Grenzziehungen werden bedroht?), sondern auch nach den kulturellen Einhegepraktiken, mit denen diesem begegnet wird. Außerdem gerät die moralische Ordnung in den Blick, die sich in der Abjektion repräsentiert und die sich nicht selten mit Stigmatisierungen und Exklusionen verwirklicht (Pfaller 2020 stellt eine Abjektanalyse des Corona-Virus vor, 2021 eine Abjektanalyse des „vierten Alters“).

## Die Beiträge in dieser Ausgabe

Anja Zimmermann nähert sich dem Abjekten in Form der *Abject Art*. In ihrem Artikel *„Everything was very neat“: Abject Art zwischen Kunstgeschichte und Politik *widmet sie sich der Rezeption von Kunstwerken, welche der *Abject Art* zugerechnet werden. Sie plädiert dafür, sich bei der Analyse der Rezeptionsgeschichte nicht nur mit der Rezeption innerhalb der Kunstgeschichte zu befassen, sondern auch die teils von moralischer Empörung getragenen Reaktionen der politischen Öffentlichkeit auf Ausstellungen oder einzelne Kunstwerke in die Aufbereitung mit einzubeziehen. Das politisch aktivierende Potenzial der *Abject Art* wird damit als inhärenter und konstitutiver Bestandteil ihrer Zuschreibungsmechanismen verstanden.

Gleich zwei Beiträge, die von Jochem Kotthaus und Hanjo Berressem, entfalten ihre konzeptionellen Überlegungen an medialen Darstellungen verstörender Praktiken der Nahrungsaufnahme.

In *Das Ende der Welt – so wie wir sie kennen. Über die Abjektion als konkrete Nihilierung des Wissens in der Moderne *liest Jochem Kotthaus Kristeva in der Tradition der Wissenssoziologie. Als zwei „Sicherungsmechanismen“ von sich verschiebenden Randbereichen des Wissens stellt er Berger und Luckmanns Konzept der Nihilierung dem der Abjektion zur Seite. Am Beispiel des Topos des Kannibalismus in *Soylent Green* und im Roman *Tender Is the Flesh* zeigt er die Wirkweise dieser Mechanismen auf: Verschiebung von unverrückbar geglaubten Grenzen, Entwertung des etablierten Wissens, Invertierung des Innen und Außen des Diskurses.

In *Fressen: Aggregatzustände des Abjekten und die Ethik im Kino *zeichnet Hanjo Berressem nach, wie Kristeva ihren Begriff des Abjekts aus der Tradition der Psychoanalyse Lacans entwickelt, indem sie entscheidende subjektkonstituierende Mechanismen konzeptionell in die Zeit vor dem Spiegelstadium verschiebt. Seine Überlegungen bebildert er mit einem ganzen Panorama an Beispielen unterschiedlicher Formen des Fressens, wie sie in dystopischen Filmen dargestellt werden (von Zombie-Kannibalismus bis zum Ekel-Essen). In der durch das Konzept der Abjektion möglich werdenden Verknüpfung der Analyse psychodynamischer wie kulturgenetischer Mechanismen zeigt er schließlich, wie die „Ethik des Kinos“ durch das Vorführen des „kulinarisch Abjekten“ im Film selbst eine Faschismus- und Kapitalismuskritik entfaltet.

In *Körper, Tod und Flüssigkeiten – Das Abjekte der Organspende* stellt Annerose Böhrer diejenigen Kulturtechniken vor, mit denen die prekär werdenden Ontologien, die sich in der Praxis der postmortalen Organspende ergeben, praktisch bearbeitet werden. Mit Kristeva beschreibt sie das Verschieben existenzieller Grenzen, die Zergliederung des Körpers, die Konfrontation mit dem Körperinneren sowie die Nähe von Tod und Sterben als Abjekte der Organspende. Schließlich deutet Böhrer das Hervorbringen von auf die Metaphern des „Geschenk des Lebens“ fokussierenden Informationsbroschüren und neutral anmutender Organspendeausweise, kultursoziologisch mit Bernhard Giesen als kulturelle Einhegepraktiken und Ordnungsversuche des Ambivalenten und Indifferenten.

Monika Mueller wählt ein historisches Beispiel. Unter dem Titel *„Look down and see my plague sores which I spread before thee my saviour“: Abjektion und Gender im puritanischen Neuengland* stellt sie an literarischen Beispielen des 17. Jahrhunderts heraus, wie sich patriarchale Machtstrukturen auch dadurch reproduzieren, dass Frauen und Männern nicht nur ungleiche Ressourcen zur Verfügung standen, um Grenzüberschreitungen auszuleben, sondern sie auch in unterschiedlichem Maße von den sozialen Mechanismen der Abjektion betroffen waren. Insgesamt führt Mueller hier die historische Reichweite der Theorie Kristevas als Kulturtheorie der westlichen Welt vor.

Auch dem Genderthema, jedoch ganz in der Gegenwart, widmet sich Gregor Balkes Beitrag *Komik mit Abjekten. Weibliche Fäkal- und Menstruationskomik in neuen TV-Serien*. Balke stellt darin nicht nur den Witz als Bearbeitungsstrategie des Abjekten vor, sondern deutet das Aufkommen des Femininen in komödiantischen Bearbeitungen von Körperausscheidungen in popkulturellen Formaten als Selbstermächtigung weiblicher Akteure. Das Abjekte wird hier nicht dem Weiblichen zugeschrieben; vielmehr erweist es sich als machtvolles Element einer Subversion der Geschlechterordnung.

In *Abjektion und existenzielle Krise* plädiert Tobias Schramm für eine präzise Bestimmung der theoretischen Konzeption der Abjektion bei Kristeva. Er führt hierfür mit Bezug auf Kant den Begriff einer existenziellen Ungewissheit ein. So sei Abjektion nicht einfach mit Prozessen des Ausschlusses und der Exklusion gleichzusetzen (was nicht selten allzu schnell geschieht). Vielmehr ist Abjektion als Moment der Herstellung von Grenzen angelegt, die einen Ausschluss erst möglich machen. Die Irritation ontologischer Grenzen versetzt das Subjekt in einen Zustand existenzieller Angst, der nur durch die (Wieder‑)Herstellung von Grenzen, die Abjektion, wieder behoben werden kann. Schramm versteht das Abjekt damit nicht als angst- oder ekelerregendes Objekt, sondern als Zustand der Ungewissheit.

Jürgen Straub führt in seinem Beitrag *Welterschließende Affekte und das explanative Potenzial eines psychoanalytischen Konzepts für die Kulturpsychologie: Abjektion* diese als heuristisch wertvolles Konzept einer Kulturpsychologie ein, welche die emotionale Verfasstheit des menschlichen In-der-Welt-Seins nicht nur anerkennt, sondern konzeptionell auf ihr aufbaut*.* Am Beispiel des Erlebens des Fremden führt er das analytische Potenzial der Abjektion vor, welche durch Abwertung und Entwertung des Fremden ordnungsstiftende und selbstbestärkende Funktionen übernimmt, gleichzeitig aber auch ein enormes destruktives Potenzial entfaltet. Um diesem entgegenzuwirken und eine pluralistische und tolerante Gesellschaft zu bestärken, bedarf es, so Straub, einer aufwendigen Gefühlsarbeit im Rahmen einer „psychologischen Aufklärung“.

Der Frage nach dem Umgang mit dem Abjekten und der Überwindung der mit Prozessen der Abjektion verbundenen Mechanismen der Exklusion und Stigmatisierung widmen sich auch Sandra Kurkowski und Maria Heckel in ihrem Beitrag *„Das könnte ich nicht“ – Abjekte in der Hospiz- und Palliativversorgung*. Mit dem *Total-pain-Modell* und der *compassionate community* stellen sie etablierte Konzepte vor, mit denen den Herausforderungen der Versorgung am Lebensende im professionellen Alltag begegnet wird. Diese professionellen Haltungen in der Hospiz- und Palliativversorgung lassen sich, so zeigen die Autorinnen, als Bearbeitungsstrategien des Abjekten analysieren und generalisieren.

Die Beiträge werfen ein Licht auf verschiedene Spannungslinien einer theoretischen Einordnung des Abjekten, auf empirische Anknüpfungspunkte und abjekte Phänomene und loten die politische Dimension (Fremdenhass, Faschismus, Pervertierungen des Kapitalismus) der Abjektion aus. Mit Bezug auf Kristeva (1982), Bataille (1999/1930) oder Lacan (1973, 1990) zeigen sie unterschiedliche kategoriale Bestimmungen des Abjekts und verschiedene Lesarten der Theorie des Abjekten auf. Sie geben einen Einblick, in welchem theoretischen Instrumentarium Abjekt und Abjektion in der jeweiligen Disziplin ihr Zuhause finden, welche theoretischen Anschlüsse sich finden lassen und wie sich andere Begriffe und Konzepte zum Abjekt ins Verhältnis setzen lassen – wie etwa der Ekel (Menninghaus 2003), existenzielle Angst und Ungewissheit, die Psychoanalyse Freuds (1955/1917, 1919, 1925) und Lacans (1973, 1990), die Kultur- und Wissenssoziologie (Giesen 2010; Berger und Luckmann 1969) oder die Gender Studies (Bovenschen 1997; Cixous 2013). Zuletzt liefern die vorgelegten Aufsätze vielfältige Hinweise auf eine empirische Umsetzung der Abjektanalyse, einer „Empirie der Abjektion“ (Biebert und Schetsche 2016, S. 119), die sich an die Theorie der Abjektion anschließen sollte.
